# Supplementary material for: Distinctive gene and protein characteristics of extremely piezophilic Colwellia
Source: BMC Genomics. 2020 Oct 6;21:692. doi: 10.1186/s12864-020-07102-y (PMC7542103; doi:10.1186/s12864-020-07102-y)
Supplement: Supplementary file 2 — Additional file 2. [file 12864_2020_7102_MOESM2_ESM.docx]

**Distinctive Gene and Protein Characteristics of Extremely Piezophilic *Colwellia***

Logan M. Peoples^1^, Than S. Kyaw^1^, Juan A. Ugalde^2^, Kelli K. Mullane^1^, Roger A. Chastain^1^, A. Aristides Yayanos^1^, Masataka Kusube^3^, Barbara A. Methé^4^, Douglas H. Bartlett^1*^

Supplementary Information

Supplementary Figure 1. Growth curves of strains of *Colwellia* *psychrerythraea* as a function of pressure and temperature. A, *C. psychrerythraea* 34H; B, *C. psychrerythraea* ND2E; *C. psychrerythraea* GAB14E.

Supplementary Figure 2. Ribosomal 16S RNA gene tree of members of the *Colwellia*. Strains in bold were compared in this study; blue are piezophilic, black are piezosensitive.

Supplementary Figure 3. Strains of *Colwellia* as a function of (A) their genome % GC, (B) full length 16S rRNA gene % GC, and (C) genome size.

Supplementary Figure 4. Isoelectric point (pI) bias within each strain. A; Isoelectric point (pI) distribution within each of the seven compared strains of *Colwellia*, colored by genome. B; Isoelectric point bias of proteins plotted as a function of % GC content within the genomes of members of the *Colwellia*, *Psychromonas*, and *Shewanella*. C; pI bias of members of the *Shewanella* plotted as a function of % GC when taking into account within-genus position broadly based on a phylogenetic tree generated by Alex & Antunes, 2019. Strains are organized by general clade grouping, labeled here as clades 1-4.

Supplementary Figure 5. Isoelectric point bias of members of the *Colwellia* as a function of optimum pressure of growth (A), depth of collection (B), or optimum temperature (C).


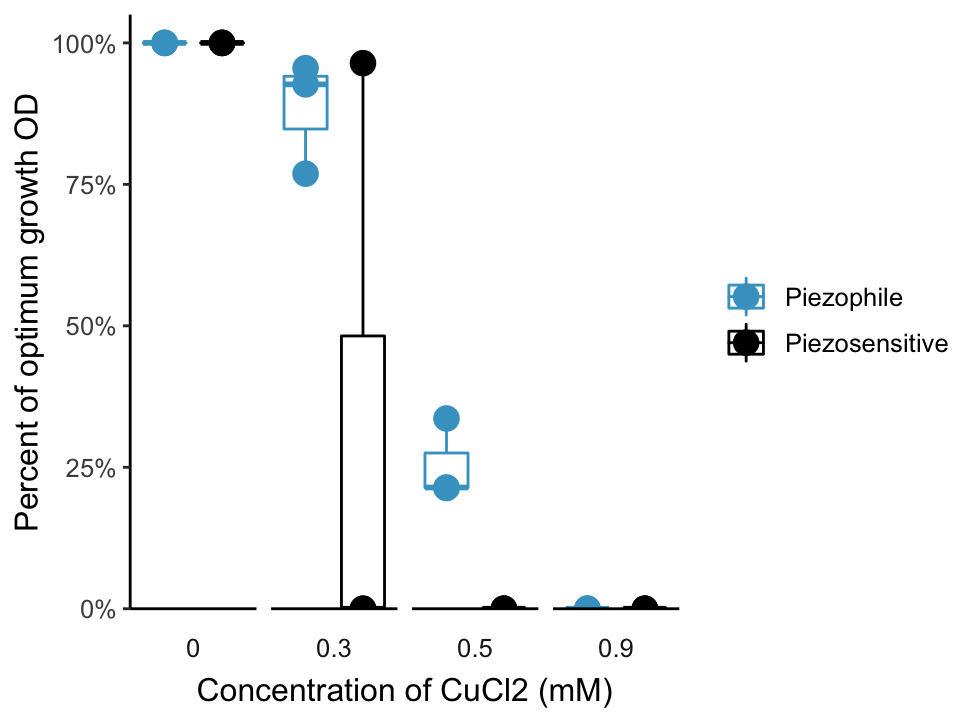


Supplementary Figure 6. Growth of strains of *Colwellia* (piezophile, n=3; piezosensitive, n-=3) measured as optical density (OD600) as a function of copper (II) chloride dihydrate concentrations at 4°C and optimum pressure. The OD values are plotted as a percentage of the OD measured under optimal conditions, when no copper (II) chloride is added.

Supplementary Figure 7. Locations of specific genomic elements identified within *Colwellia* *marinimaniae* MT41.
